# Expression of interferon-inducible chemokines and sleep/wake changes during early encephalitis in experimental African trypanosomiasis

**DOI:** 10.1371/journal.pntd.0005854

**Published:** 2017-08-18

**Authors:** Claudia Laperchia, Chiara Tesoriero, Paul F. Seke-Etet, Valentina La Verde, Valeria Colavito, Gigliola Grassi-Zucconi, Jean Rodgers, Paul Montague, Peter G. E. Kennedy, Marina Bentivoglio

**Affiliations:** 1 Department of Neuroscience, Biomedicine and Movement Sciences, University of Verona, Verona, Italy; 2 Department of Diagnostics and Public Health, University of Verona, Verona, Italy; 3 Institute of Biodiversity, Animal Health & Comparative Medicine, University of Glasgow, Glasgow, United Kingdom; 4 Institute of Infection, Immunity & Inflammation, University of Glasgow, Glasgow, United Kingdom; 5 National Institute of Neuroscience (INN), Verona Unit, Verona, Italy; Makerere University, UGANDA

## Abstract

**Background:**

Human African trypanosomiasis or sleeping sickness, caused by the parasite *Trypanosoma brucei*, leads to neuroinflammation and characteristic sleep/wake alterations. The relationship between the onset of these alterations and the development of neuroinflammation is of high translational relevance, but remains unclear. This study investigates the expression of interferon (IFN)-γ and IFN-inducible chemokine genes in the brain, and the levels of CXCL10 in the serum and cerebrospinal fluid prior to and during the encephalitic stage of trypanosome infection, and correlates these with sleep/wake changes in a rat model of the disease.

**Methodology/Principal findings:**

The expression of genes encoding IFN-γ, CXCL9, CXCL10, and CXCL11 was assessed in the brain of rats infected with *Trypanosoma brucei brucei* and matched controls using semi-quantitative end-point RT-PCR. Levels of CXCL10 in the serum and cerebrospinal fluid were determined using ELISA. Sleep/wake states were monitored by telemetric recording. Using immunohistochemistry, parasites were found in the brain parenchyma at 14 days post-infection (dpi), but not at 6 dpi. *Ifn-*γ, *Cxcl*9, *Cxcl10* and *Cxcl11* mRNA levels showed moderate upregulation by 14 dpi followed by further increase between 14 and 21 dpi. CXCL10 concentration in the cerebrospinal fluid increased between 14 and 21 dpi, preceded by a rise in the serum CXCL10 level between 6 and 14 dpi. Sleep/wake pattern fragmentation was evident at 14 dpi, especially in the phase of wake predominance, with intrusion of sleep episodes into wakefulness.

**Conclusions/Significance:**

The results show a modest increase in *Cxcl*9 and *Cxcl11* transcripts in the brain and the emergence of sleep/wake cycle fragmentation in the initial encephalitic stage, followed by increases in *Ifn-*γ and IFN-dependent chemokine transcripts in the brain and of CXCL10 in the cerebrospinal fluid. The latter parameter and sleep/wake alterations could provide combined humoral and functional biomarkers of the early encephalitic stage in African trypanosomiasis.

## Author summary

Human African trypanosomiasis, also known as sleeping sickness, is caused by infection with the parasite *Trypanosoma brucei*. Although the number of reported cases has declined in recent years, the disease still represents a challenge. During the second, meningoencephalitic, stage of the disease trypanosomes invade the brain. Consequently the infection can only be cured with toxic drugs which enter the brain, and is fatal if left untreated. Identifying the onset of this stage is therefore of crucial importance for therapeutic decisions. Using experimental infection in rats, we investigated the expression of interferon-γ and interferon-dependent chemokine genes involved in the disease progression. In parallel, we determined when the characteristic alterations of the sleep/wake cycle emerge in relation to the onset of the encephalitic stage, which was assessed histologically by the detection of parasites in the brain tissue. The results indicate that detection of perturbation of the sleep/wake cycle, especially the intrusion of sleep episodes into wakefulness, followed by an increase in CXCL10 concentration in the cerebrospinal fluid, could provide combined functional and humoral biomarkers of the early encephalitic stage of African trypanosomiasis.

## Introduction

Human African trypanosomiasis (HAT) is a neglected tropical disease that is still endemic in foci in rural regions of sub-Saharan Africa. The disease is caused by infection with specific subspecies of *Trypanosoma brucei (T*. *b*.*)*. *T*. *b*. *gambiense* is found in West and central Africa and causes a chronic form of the disease, and *T*. *b*. *rhodesiense* found in East Africa causes a more acute form. *T*. *b*. *gambiense* HAT accounts for over 95% of reported cases [1], with reported case numbers declining considerably in recent years [2, 3]. The WHO's aim is to eliminate HAT as a public health problem with a target date of 2020, and to achieve a complete interruption of HAT transmission with a a target date of 2030 [4]. This aim is supported by sustained surveillance [3], but is facing considerable challenges [1]. Concerns are being raised about the viability of eliminating the disease within the WHO time-frame due, among other issues [1], to underreporting [5], and to the finding of *T*. *b*. *gambiense* reservoirs in skin or adipose tissue of asymptomatic individuals [6].

Early after *T*. *b*. transmission, which occurs through bites of the insect vector (tsetse flies of the genus *Glossina*), the parasites invade the hemolymphatic system and peripheral organs. This first, hemolymphatic, stage can be effectively treated with Suramin or Pentamidine, drugs that have been in use for over 50 years. If the disease remains untreated, the parasites enter the CNS and the infection progresses to the second, meningoencephalitic, stage. A wealth of evidence points to *T*. *b*. traversal of the blood-brain barrier (BBB) as the key pathogenetic event leading to this second stage [7]. Alternative or additional mechanisms of brain infection, based on parasite diffusion via a meningeal route and/or the blood-cerebrospinal fluid (CSF) barrier and/or perivascular spaces, have also been proposed [8, 9]. Clinically, the disease evolves into a complex neuropsychiatric syndrome with disruption of sleep/wake patterns [10–12] that gave HAT the popular name of sleeping sickness.

Due to the nonspecific clinical signs and symptoms of the hemolymphatic stage of HAT, it is likely that most patients present in the meningoencephalitic stage, which, if left untreated, is almost always fatal [11]. The trypanocidal drugs used to cure the first stage of HAT poorly cross the BBB, and toxic drugs are instead widely used for the treatment of the second stage [11]. Staging of HAT and determining viable disease biomarkers thus represent key issues with obvious therapeutic implications. Staging of HAT is currently based on criteria indicated by WHO [4] related to the presence of parasites and/or counts of white blood cells in the CSF. However, such criteria lack sensitivity and therefore the identification of new staging tools is of critical importance [11–13].

In humans and in rodent models, *T*. *b*. infection results in a neuroinflammatory pathology with regional microglia activation [14, 15] and experimental data have indicated that T-cell recruitment to the brain paves the way for parasite entry [7]. Pro- and anti-inflammatory mediators, in particular the chemoattractant chemokines CXCL10 and CXCL13, have been investigated as candidate of CSF biomarkers for the encephalitic stage of *T*. *b*. infection [16]. Combined panels of neuroinflammatory markers have been proposed for *T*. *b*. *gambiense* [17, 18] and *T*. *b*. *rhodesiense* [19] HAT staging. In these panels, CXCL10, an interferon (IFN)-inducible chemokine, was especially effective as a potential marker for the CNS disease, and has been proposed to represent an early indicator of CNS involvement in HAT [19].

Experimental data have pointed to a pivotal role of IFN-γ released from activated T-cells in *T*. *b*. traversal of the BBB [7, 20], and of CXCL10 in facilitating the accumulation of T-cells in the neuropil [16]. Invasion of the cerebral parenchyma by African trypanosomes has been shown to occur over time, after an initial interval following peripheral infection [7, 21]. However, the temporal relationship of trypanosome neuroinvasion with neurological signs and symptoms, of crucial importance to facilitate optimal management of HAT patients [19], requires further investigation.

Sleep/wake alterations in HAT are represented by two main changes [22, 23], documented also in rat models of the infection [24, 25]. These alterations include sleep/wake cycle disruption, with episodes of daytime somnolence and nocturnal insomnia, and alterations of the structure of sleep. Recent experimental data have indicated that the changes in sleep structure can precede parasite neuroinvasion and cannot, therefore, provide a biomarker of the onset of the encephalitic stage [21]. The emergence of sleep/wake cycle fragmentation in relation to parasite entry into the brain parenchyma remains to be assessed.

On this basis, the present experimental study aimed to clarify the relationship between altered levels of IFN-inducible chemokines, in particular CXCL10, and changes of the sleep/wake cycle during *T*. *b*. infection. Using a rat model and histological assessment of parasites within the brain parenchyma, the expression of genes encoding *Ifn-*γ and the IFN-inducible chemokines *Cxcl9*, *Cxcl10*, *Cxcl11* in the brain was here evaluated, CXCL10 levels were measured in the serum and CSF, and sleep/wake parameters were investigated in parallel.

## Materials and methods

### Ethics statement

Animal handling and surgery were performed following the “Principles of laboratory animal care” (NIH publication No. 86–23, revised 1985), with a protocol approved by the Animal Care and Use Committee of the University of Verona (CIRSAL) and authorized by the Italian Ministry of Health (protocol n°18/2012-B), in strict adherence to the European Communities Council (86/609/EEC) directives and the ARRIVE guidelines, minimizing the number of animals used and avoiding their suffering.

### Animals and infection

Adult (3–6 month-old) male Sprague-Dawley rats were purchased from Harlan Laboratories (Milan, Italy) and housed in the animal facilities at the Medical School of the University of Verona under veterinarian control and standard environmental conditions of temperature and humidity and a 12h/12h light/dark cycle (lights on at 7 am, corresponding to *Zeitgeber* time, ZT, 0), with free access to food and water.

Rats were infected by intraperitoneal (ip) injection with pleomorphic *T*. *b*. *brucei* parasite strain An Tat 1/1 (100 parasites/g body weight) derived from stabilate EATRO 1125 kindly provided by the Laboratory of Serology, Institute of Tropical Medicine Prince Leopold, Antwerp, Belgium). Parasitaemia was assessed at 3 and/or 5 days post-infection (dpi) in a blood sample from the tail vein.

### Experimental design

In this model, animal death occurs at 4–5 weeks post-infection [26]. On the basis of our recent study [21], 6 dpi was here used as time point preceding parasite neuroinvasion, and 14 dpi as time point corresponding to initial parasite neuroinvasion, as also here verified histologically. Later time points (19, 21, 30 dpi) were used for analyses during the progression of the meningoencephalitic stage.

Infected rats and matched uninfected controls were randomly destined for the quantification of CXCL10 in the serum and CSF by enzyme-linked immunosorbent assay (ELISA) at different post-infection time points (Table 1). The brains of these animals, as well as those of matched animals, were destined for the analysis of the expression of *Ifn-*γ and IFN-inducible chemokine genes or immunohistochemical verification of the occurrence of parasites in the brain parenchyma at 6 and 14 dpi (Table 1). The rats used for these analyses were not implanted with probes for telemetric recording to avoid any confounding factor due to a potential inflammatory reaction. Additional animals were therefore used for continuous telemetric monitoring of sleep/wakes states before and after the infection (Table 1).

**Table 1:** Rats destined for each analysis.

Sampling	ELISA		PCR	IHC	EEG/ EMG
	Serum	CSF			
**ctrl**	n = 7	n = 5	n = 3		n = 6^+^
**6 dpi**	n = 4	n = 3	n = 3	n = 4	
**14 dpi**	n = 9	n = 6	n = 3	n = 5	
**19 dpi**					n = 5^**§**^
**21 dpi**	n = 7	n = 4	n = 3		
**30 dpi**	n = 5	n = 3			

^+^ Recording over time

^**§**^ Archival data

Abbreviations: CSF, cerebrospinal fluid; ctrl, control (uninfected); dpi, days post-infection; EEG/EMG, electroencephalography/electromyography (telemetric recording for sleep/wake analyses); IHC, immunohistochemistry (blood vessel wall and parasite detection)

### PCR analysis

The levels of *Ifn*-*γ*, *Cxcl9*, *Cxcl10* and *Cxcl11* transcripts relative to the activity of the housekeeping gene *cyclophilin* (*Cyc*) were determined in the brain using end-point RT-PCR at 6, 14, 21 dpi and uninfected controls (Table 1). As described elsewhere [27], total cellular brain RNA was prepared by homogenization in RNA-Bee-(amsbio-UK). Random hexamer primed cDNA synthesis was performed on 2 μg RNA using SuperScript III (Invitrogen-UK). End-point RT-PCRs were completed using RedTaq Ready Mix (Sigma-UK) in a total reaction volume of 25 μl containing 5 μl cDNA and 0.3 μM primers against the specific gene transcripts (Table 2). All PCRs were performed in the linear amplification range over 25–30 amplification cycles (Table 2) comprising an initial denaturation step of 94°C/5 mins, a core cycle comprising (94°C/1 min−55°C−65°C/1 min−72°C/1 min) followed by a final extension of 72°C/10 mins [27]. PCR products were separated by TAE gel electrophoresis, visualised by ethidium bromide staining and quantified by densitometry using an UVIdocD55XD documentation system (Uvitec UK).

**Table 2:** Primer sequences, product length and amplification cycles performed in end-point PCR analysis.

Gene	Forward Primer (5^’^>3^’^)	Reverse Primer (5^’^>3^’^)	CycleNo.	Product length
*Cyc*	ACCCCACCGTGTTCTTCGAC	CATTTGCCATGGACAAGATG	25	300bp
*Ifn-γ*	CAAGGCACACTCATTGAAAGCCTA	TTATTGGCACACTCTCTACCCCAGA	30	431bp
*Cxcl9*	TGAAGTCCGTTGCTCTATTCCTCA	TTAGATGCAGAGCGCTTGTTGGTA	25	411bp
*Cxcl10*	CCTGCATCGACTTCCATGAACAGA	TGGGGCATGGCACATGCTGA	25	520bp
*Cxcl11*	GTGAAAGTGGTCAAAATGGCAGCA	ATGTGCCTCGTGTTATTTGGGGAA	25	520bp

### CXCL10 analyses

Samples of CSF, blood, and brain were collected at different times after infection and in matched uninfected rats (Table 1). The rats were deeply anesthetized by ip injection of pentobarbital (0.05 mg/g body weight), and CSF was collected by the insertion of a borosilicate glass capillary tube in the cisterna magna through the dura mater, as previously described [28]. CSF samples with blood contamination were excluded from the study. Blood samples were collected by cardiac puncture, allowed to clot for 2 h at room temperature, and centrifuged for 20 minutes at 2000 x g. The collected serum samples were aliquoted, stored at -20°C and subsequently analyzed by an ELISA assay. Immediately after CSF and blood collection, the anesthetized rats were sacrificed by cervical dislocation. The brains were excised, frozen in liquid nitrogen, and stored at -80°C until processing for PCR or immunohistochemistry (Table 1).

The volume of CSF samples was sufficient for the analysis of only one molecule and CXCL10 was selected for the analyses on the basis of previous results in a murine model of the disease and in the CSF of *T*. *b*. *gambiense* HAT patients [16]. The levels of CXCL10 in the serum and CSF were quantified using the Quantikine Mouse CXCL10/IP-10/CRG-2 immunoassay by R&D Systems (Milan, Italy). Since rat ELISA kits are not commercially available, a mouse CXCL10 (IP10; CRG2) with 80% affinity for rat CXCL10, previously validated for measurement of CXCL10 levels in the rat serum [29], was used. Samples were processed according to the supplier’s instructions. Serum samples were analyzed in triplicate, while CSF samples were analyzed in single or duplicate due to volume constraints. The optical density (OD) reading at 450 nm was corrected for optical imperfection in the plate. The data were linearized by plotting the log of the mouse CXCL10 concentrations *versus* the log of the OD and the best fit line was determined by regression analysis.

### Immunohistochemical procedure and confocal microscopy

Frozen brains collected as above were used for immunohistochemistry (Table 1). Coronal sections were cut at a 20 μm thickness using a cryostat, collected in series, air dried and fixed in cold acetone at 4C° for 10 min prior to staining. The sections were pre-incubated in a solution of 2% normal donkey serum and 0.3% Triton X-100 in 0.1M phosphate buffer, pH 7.4. The sections were then incubated overnight at 4°C in a mixture of primary antibodies to visualise blood vessel walls and parasites. Blood vessel walls were immunolabeled using goat polyclonal anti-glucose transporter (GLUT)-1 antibodies (1:100; Santa Cruz Biotechnology, Santa Cruz, CA, USA), and parasites with rabbit polyclonal antibodies which recognize the *T*. *b*. variant surface glycoprotein of the AnTat 1:1E stabilate (1:500; kindly supplied by the Institute of Tropical Medicine, Antwerp, Belgium). After rinsing in PBS, the sections were incubated in a solution of secondary antibodies containing Alexa Fluor 488-conjugated donkey anti-goat IgGs and Alexa Fluor 568-conjugated donkey anti-rabbit IgGs (InVitrogen Corporation, Carlsbad, CA, USA; 1:1000). The sections were then rinsed in PBS, and mounted with using Dako mounting medium (Dako, Hamburg, Germany).

Images were acquired with the confocal microscope Leica SP5 (Leica, Manheim, Germany). Serial Z-planes (1.8 μm) images were captured with the Leica Application Suite software, and collapsed into a single image to which colors were assigned. Lateral views were obtained using Imaris 7.4 software (Bitplane, Zurich, Switzerland) after optimization of contrast and brightness.

### Telemetric recording and electroencephalogram (EEG) analysis

For neurophysiological analyses, surgical implantation of the radiotelemetric probes and recordings of EEG and electromyography by radiotelemetry (Data Science International [DSI], St. Paul, MN, USA) were performed under deep anesthesia (20 mg/kg i.p. Zoletil (tiletamine +zolazepam, Virbac, Cédex, France). Following baseline recordings, performed 10 days after surgery, the rats were infected as described above. Thereafter, recordings were continued until 14 dpi, when the rats were anaesthetized and killed by cervical dislocation.

Data obtained at 6 and 14 dpi *versus* baseline were analyzed using MatLab software (The MathWorks, Natick, MA, USA). EEG and electromyography signals were visually scored for 10 s epochs. Four different states—wakefulness (W), slow wave sleep (SWS), rapid eye movement (REM) and sleep-onset REM sleep (SOREM)—were distinguished according to standard criteria in rodents [30–32]. SOREM episodes are a sleep structure alteration in which, instead of the normal SWS-REM sleep sequence, REM sleep is preceded by W. According to a previous study[31], SOREM episodes were defined as a period of at least 40 s of W followed by REM sleep events spanning 10 s or more. The mean REM sleep latency, which was calculated as previously described [33], and the other vigilance state parameters were analyzed for a 24h (ZT0-ZT24) period. In addition, a hypnogram at 19 dpi was derived from archival material collected during a previous study [25] for a qualitative comparison with the present data.

### Statistics

PCR data was investigated by general linear model analysis followed by Tukey’s *post-hoc* test using Minitab Version 17 (Minitab Inc, State College, Pennsylvania, USA.) software.

Statistical analyses of ELISA and EEG data were performed using the SPSS software (Chicago, Illinois, USA). For the ELISA data, comparisons between the control and infected groups as well as between different time points were evaluated with one-way analysis of variance (ANOVA), followed by the Tukey *post-hoc* test. For the EEG data, comparison with the baseline values were carried out using one-way repeated measure ANOVA, followed by the Bonferroni *post-hoc* test for pairwise comparison.

Data are presented as mean ± standard error of the mean (SEM). In all the analyses, statistical significance was set at p ≤ 0.05.

## Results

### Expression of inflammation genes

**Fig 1:**
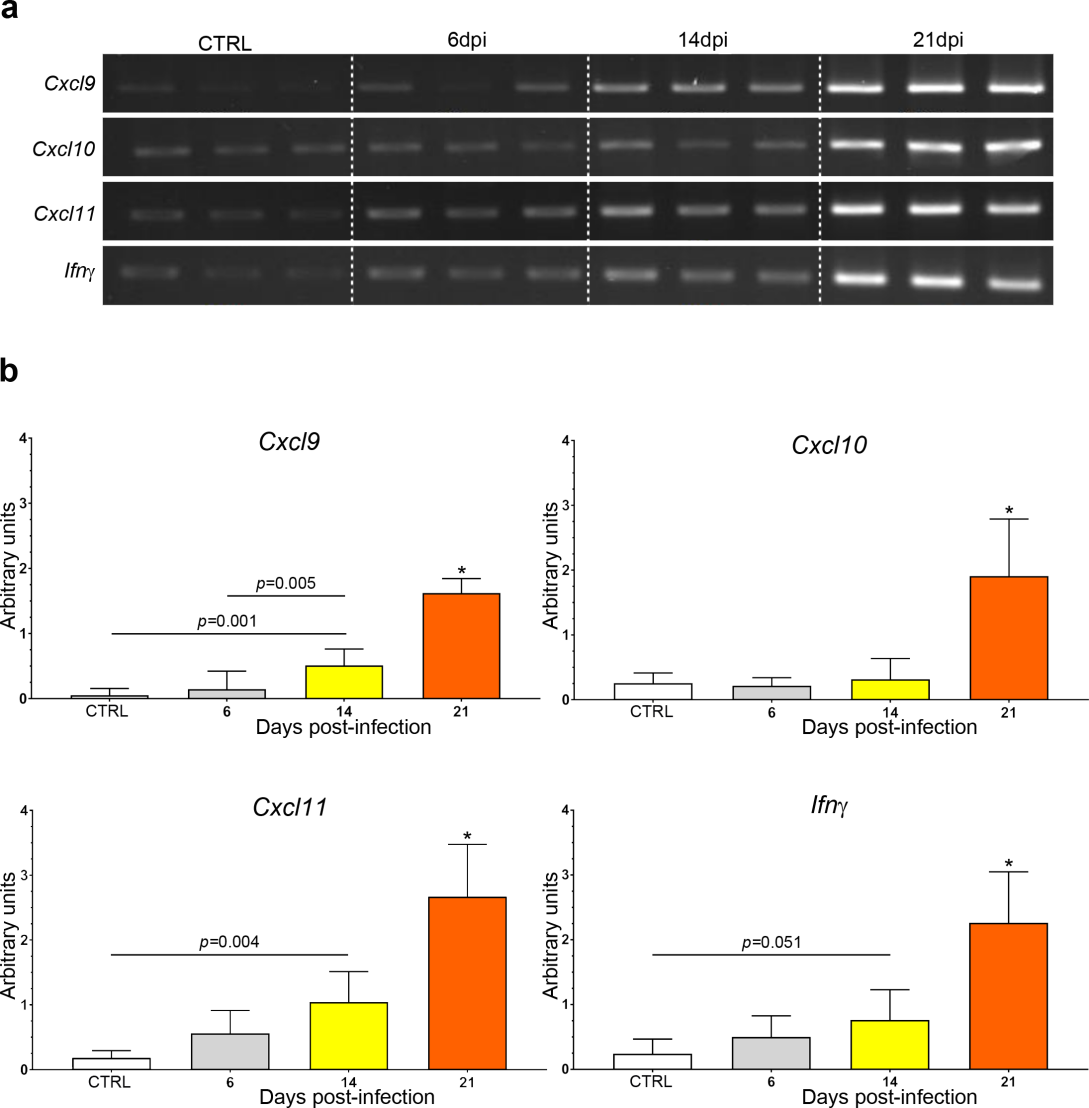
mRNA levels of *Ifn-*γ and IFN-dependent chemokines in the brain following *T*. *b*. *brucei* infection. (A) PCR reaction products from triplicate samples for each time point were electrophoresed through a single 2% agarose gel and the corresponding band intensities quantified using an Uvitec gel documentation system. A progressive increase in signal strength can be seen as the infection progresses. (B) Graphs display a summary of the densitometry data, normalised against *Cyclophilin* expression, and resulting statistical analysis. Data are expressed as the group mean and 95% confidence interval. n = 3 in all groups. *significantly (*p*<0.001) higher gene expression levels were detected at 21 dpi compared with all other time points. Horizontal lines indicate a significant difference between group means. Corresponding *p*-values are given above the line.

End-point RT-PCR analysis revealed a common temporal expression profile of the four genes during disease progression (Fig 1A) although there was some degree of inter-gene variation in message levels (Fig 1B). No changes were apparent at 6 dpi. However, a modest, though significant upregulation in *Cxcl*9 (0.511 ± 0.058, p = 0.001) and *Cxcl11* (1.044 ± 0.109, p = 0.004) expression was detected in rats at 14 dpi compared with uninfected controls (0.054 ± 0.024, 0.182 ± 0.026, respectively). An increase in the expression of *Ifn-*γ (0.765 ± 0.108) was also noted at 14 dpi compared to uninfected controls (0.244 ± 0.052) but this failed to reach statistical significance (p = 0.051). A further approximately three-fold increase (p<0.001) in the expression of the *Ifn-*γ, *Cxcl*9 and *Cxcl11* genes (2.264 ± 0.182, 1.623 ± 0.051, 2.671 ± 0.187 respectively), was found between 14 and 21 dpi.

No significant changes were found in the *Cxcl10* mRNA levels during the initial time points. However, expression of this gene was significantly elevated (p<0.001), by six-fold or greater, at 21 dpi (1.911 ± 0.204) compared with levels seen at day 0 (0.259 ± 0.036), 6 dpi (0.217 ± 0.029) and 14 dpi (0.318 ± 0.74). Summary statistics are provided in Supplementary Table 1.

### CXCL10 levels in the serum and CSF during the progression of the infection

**Fig 2:**
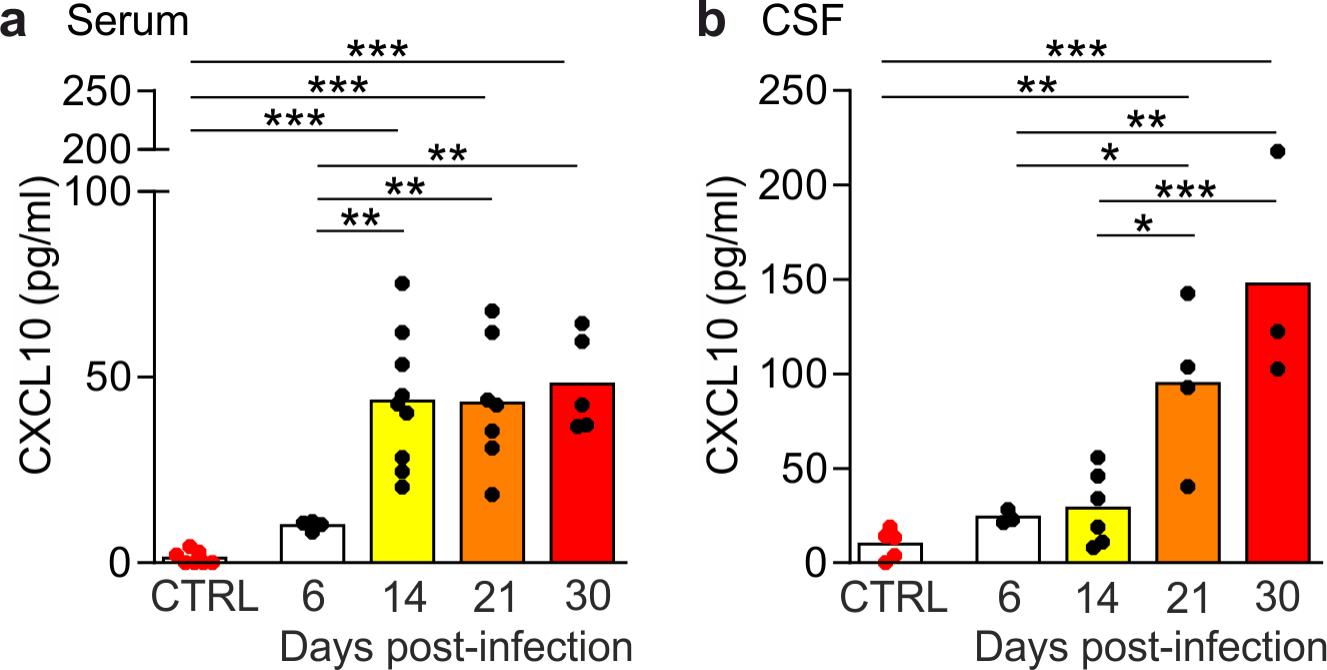
CXCL10 levels increase in serum and CSF of *T*. *b*. *brucei*-infected rats during disease progression. (A) In the serum, a steep increase in serum CXCL10 levels is observed between 6 (n = 4) and 14 (n = 9) days post-infection (dpi) compared with control group (n = 7), and the increase persists at 21 dpi (n = 7) and 30 dpi (n = 5). (B) In the CSF, CXCL10 levels increase significantly at 21 dpi (n = 4) and further increase at 30 dpi. Dots represent individual rats, bars indicate mean group values ± SEM (*p < 0.05, **p<0.01, ***p<0.001, Tukey *post-hoc* test following one-way ANOVA).

A progressive significant increase of CXCL10 concentration was found both in the serum (one-way ANOVA: F (4,27), p<0.0001) and in the CSF (F (4,16), p<0.0001) of *T*. *b*. *brucei*-infected rats. Interestingly, significant differences documented by *post-hoc* testing showed that the increase occurred at different time points in the two biological fluids over the analyzed course of the infection (Fig 2).

In the serum (Fig 2A), the level of CXCL10 in uninfected rats was 1.35±0.68 pg/ml. During the hemolymphatic stage of the disease (between 0 and 6 dpi), the level of the chemokine increased (10.08±0.62 pg/mol) but the difference *versus* controls did not reach statistical significance (p = 0.85). During the progression of the infection, a significant increase of CXCL10 level in the serum (43.61+5.98 pg/mol) *versus* controls (p<0.0001) and *versus* 6 dpi (p = 0.0031) was recorded at 14 dpi. Significantly higher levels of CXCL10 in the serum of infected rats *versus* controls persisted at subsequent time points (21 dpi: 43.05±6.53 pg/ml, 30 dpi: 48.13±5.84 pg/ml; p<0.0001 at both time points).

The analysis of the CSF (Fig 2B) showed that in the infected rats CXCL10 levels significantly increased above control levels (10.03±3.51 pg/ml) during disease progression (21 dpi: 94.84±21.10 pg/ml, p = 0.0062; 30 dpi: 147.6±35.54 pg/ml, p<0.0001), with a significant increase between 14 dpi (29.03±7.95 pg/ml) and 21 dpi (p = 0.030), as well as between 6 dpi (24.19±2.11 pg/ml) and 21 dpi (p = 0.055).

### Initial parasite neuroinvasion

**Fig 3:**
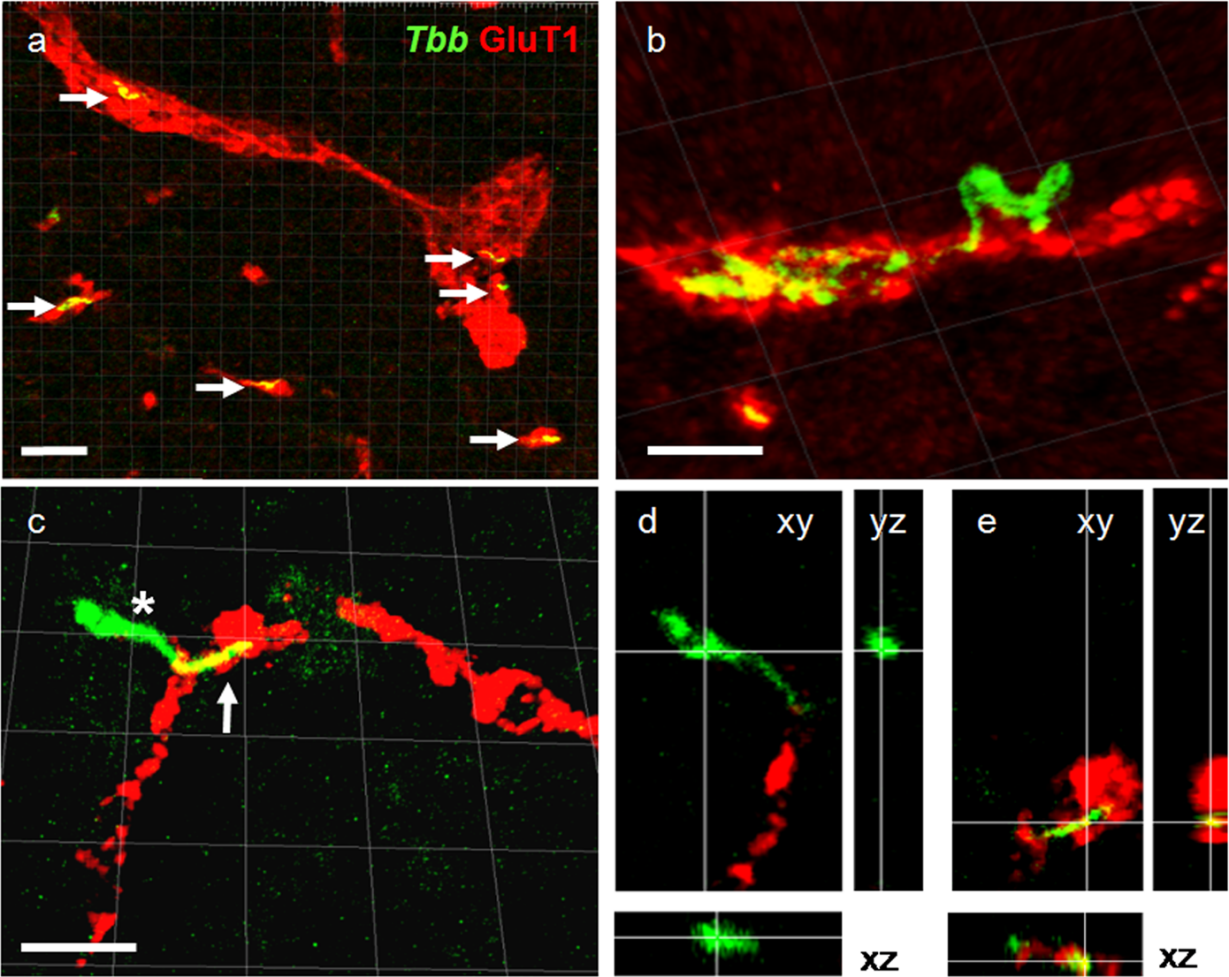
Parasites crossing blood vessels in the brain parenchyma 14 days after infection. Blood vessels (red) are visualised by-glutamate transporter 1 (GluT1) immunoreactivity, *Trypanosoma brucei brucei (Tbb*) are in green (A-E). (A) Intravascular parasites are frequently observed in the cerebral cortex. (B) An extravascular parasite crawling along the external walls of a blood vessel. Intravascular parasites are also visible. (C) Maximum intensity projection showing a parasite transmigrating with the orientation of the flagellum (arrow) that seems to indicate passage from the bloodstream to the neuropil. (D-E) Orthogonal views of a single plane (xy; yz; xz) of the parasite cell body outside the blood vessel (D, asterisk in C) and flagellum (E, arrow in C) within in the blood vessel.

No trypanosomes were detected within the brain parenchyma of rats sacrificed at 6 dpi although parasites were observed within brain capillaries and in the choroid plexus. At 14 dpi, parasites had progressed into the neuropil of all the brains examined. In addition, trypanosomes were frequently observed traversing blood vessel walls (Fig 3).

### Sleep/wake alterations

**Fig 4:**
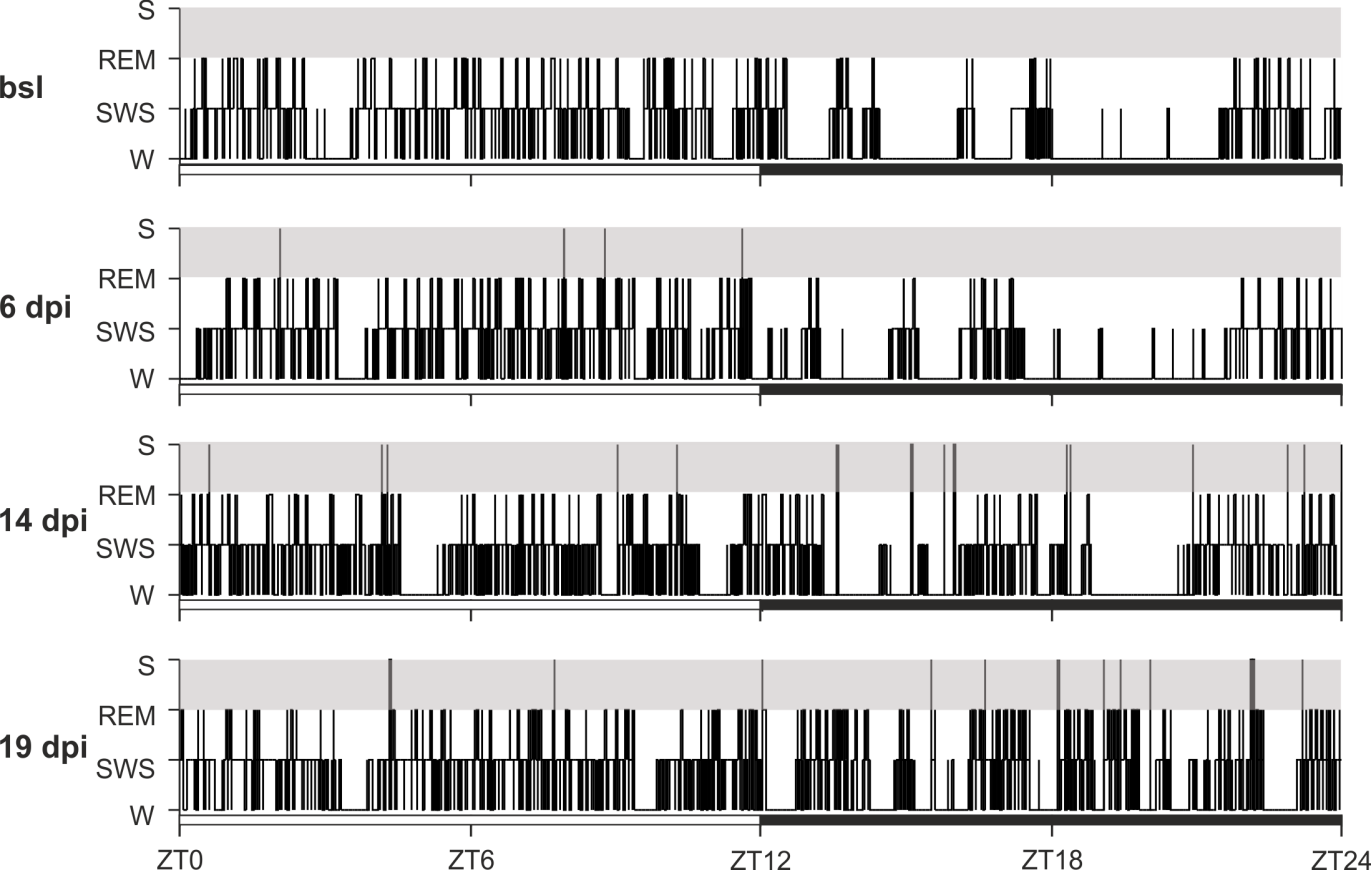
24 h hypnograms in rats recorded before and after *T*. *b*. *brucei* infection. Hypnograms shown are examples from one animal at each point studied. Note that a progressive sleep-wake fragmentation and increase in the number of SOREM episodes (S) are observed during both the light and dark phases (indicated by the bars) compared with the baseline (bsl) recorded prior to the infection. The hypnogram at 19 dpi (day post-infection) was analyzed from archival material [25]. (*Zeitgeber time*, ZT, 0 corresponds to the lights-on time).

The hypnograms at 6, 14 and 19 dpi showed a progressive fragmentation of the sleep-wake pattern, especially during the dark phase (Fig 4), in which wake was predominant in baseline recordings as expected in normal conditions in nocturnal rodents. Such fragmentation became evident at 14 dpi and was exacerbated at 19 dpi, with an increase of SOREM episodes, especially during the dark period.

**Fig 5:**
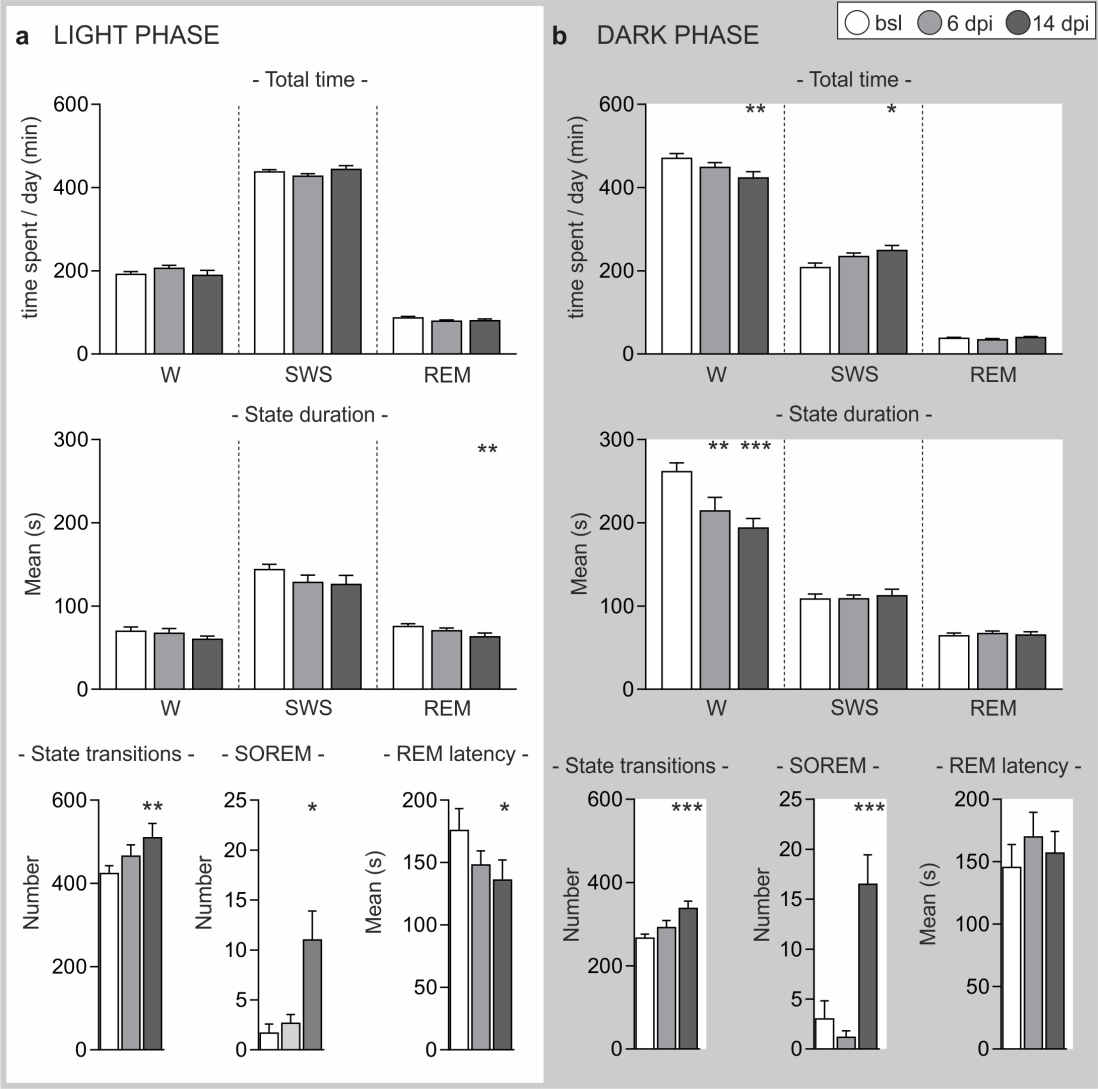
Changes in the sleep/wake pattern. Quantitative data of vigilance state parameters are presented as mean ± SEM (*p < 0.05, **p < 0.01, ***p < 0.001, Bonferroni *post-hoc* test following one-way repeated measure ANOVA). Values at 6 and 14 days post infection (dpi) are compared with baseline values (bsl, before infection) (n = 6). (A) In the light phase, the time spent in each state as well as the mean duration of W and SWS episodes is not significantly altered at 6 and 14 dpi. A significant decrease of both the mean duration of REM sleep episodes and REM latency is found at 14 dpi. At the same time point, the number of state transitions and the number of SOREM episodes increase significantly. (B) In the dark phase, the time spent in W decreases significantly at 14 dpi, whereas the time spent in SWS increases. No changes are observed in the total time spent in REM sleep. A significant decrease in the mean duration of W episodes is observed from 6 dpi. In addition, the number of state transitions and SOREM episodes increases significantly at 14 dpi.

Quantitative evaluation of the vigilance states (W, SWS, REM sleep) was pursued comparing recordings at 6 and 14 dpi with baseline recordings prior to the infection. The light and dark periods were analyzed separately. During the light period (Fig 5A), the main findings were represented by a significant decrease of the mean REM latency and of the mean duration of REM sleep episodes, as well as by a significant increase in the number of state transitions at 14 dpi. No changes in either the time spent in each state or in the mean duration of W and SWS episodes were observed at this time point.

Occasional, isolated SOREM episodes were observed before infection as also reported in healthy humans [34]. In the infected animals, 3 out of 6 rats displayed SOREM episodes during the light period at 6 dpi, which frequently occurred in clusters. By 14 dpi all rats exhibited these changes in their sleep structure.

At this point (14 dpi) the sleep/wake changes were more marked during the dark period than during the light period (Fig 5B). In particular, the total time spent in W decreased whereas the total time spent in SWS increased during the dark period. In addition, a highly significant increase in the number of state transitions was observed, accounting for the fragmentation evident in the hypnograms at 14 dpi (Fig 4). Alterations were also documented in the mean duration of W episodes, which decreased by 6 dpi, with a more marked decrease at 14 dpi. No changes in the total time spent in REM sleep or in the mean duration of SWS and REM sleep episodes were observed, nor were there any changes in the mean REM latency. SOREM episodes were observed during the dark phase, and their number significantly increased at 14 dpi (Fig 5B).

Overall, the analysis of vigilance states pointed out that sleep-wake fragmentation was already marked at 14 dpi, with rapid cycling between sleep state episodes and a progressive invasion of sleep into wakefulness in the dark period (Figs 4 and 5).

## Discussion

**Fig 6:**
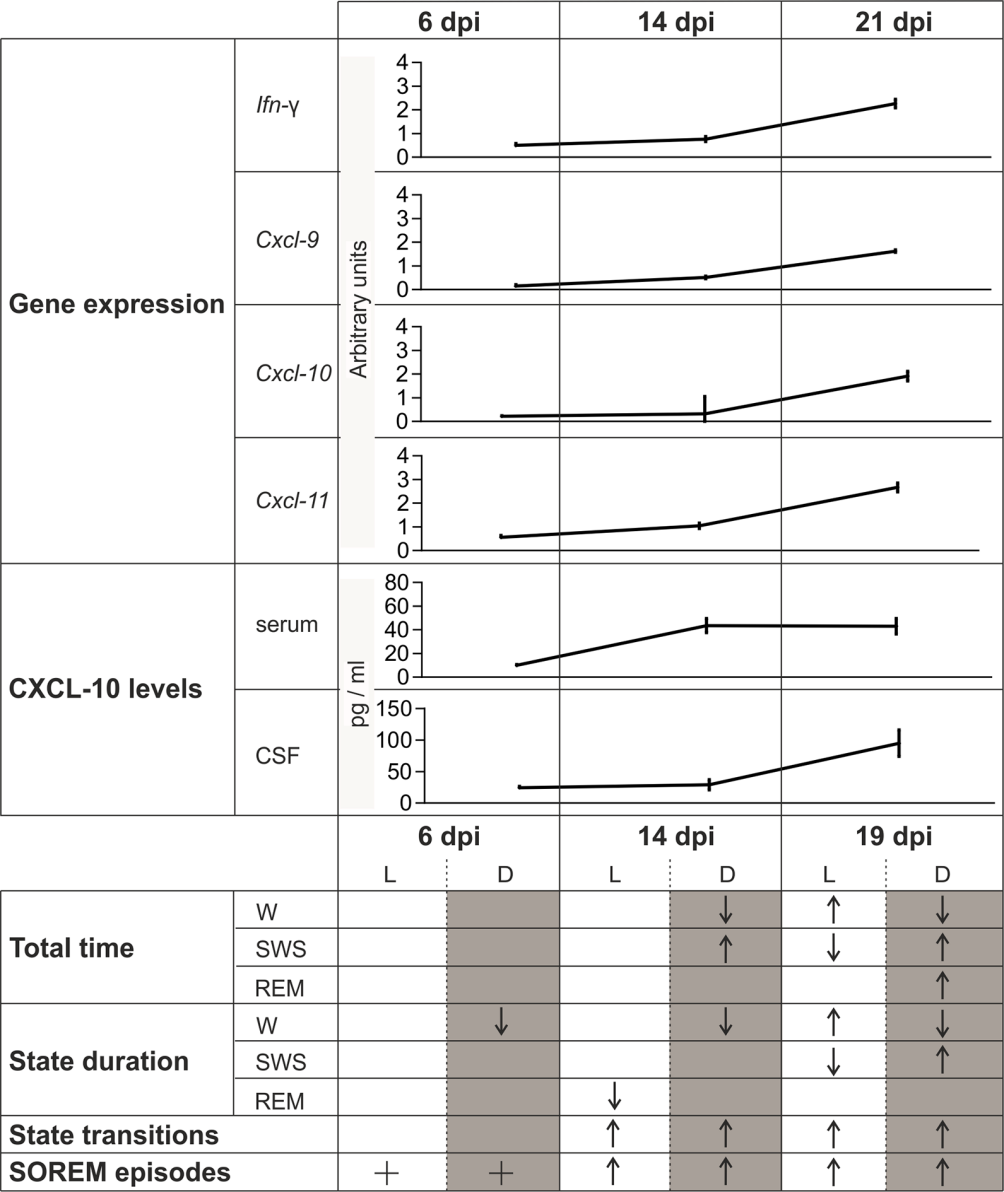
Summary of the changes in the investigated parameters during the first three weeks of infection. As assessed by parasite detection in the brain parenchyma, 6 days post-infection (dpi) corresponds to the first, hemolymphatic, stage, and 14 dpi to initial parasite neuroinvasion; 19 dpi and 21 dpi correspond to early encephalitis. The study of transcripts showed a progressive increase in the expression of *Ifn-γ*, *cxcl-9*, *cxcl-10* and *cxcl-11* mRNAs in the brain, which persisted at 21 dpi. CXCL10 increased in the serum at the time of initial parasite neuroinvasion and in the cerebrospinal fluid (CSF) during early encephalitis. Wake (W), rapid eye movement (REM) sleep, slow wave sleep (SWS) indicate the vigilance states analyzed during the light (L) and dark (D) periods. Significant increases and decreases are indicated by directional arrows; + indicates the onset of sleep onset REM episodes (SOREM). Note that an initial fragmentation of vigilance states (an increase of the time spent in SWS and of the number of state transitions, together with a decrease of the total time and the mean duration of W episodes) became evident at the time of parasite neuroinvasion (14 dpi), especially during the period of wakefulness predominance (dark phase), with intrusion of sleep episodes into wakefulness. The onset of SOREM episodes, which were observed from 6 dpi onward, preceded this event. Although a more restricted definition was used in the present study than in our previous investigation [22], a significant increase in the number of SOREM episodes was observed from 14 dpi.

The findings of this study show an increase in the levels of *Cxcl9* and *Cxcl11* mRNAs in the brain at the time of initial parasite neuroinvasion (between 6 and 14 dpi), which continued to rise during the progression of the disease, and significant increases in the levels of *Cxl10* and *Ifn-γ* mRNAs in the brain were apparent by 21 dpi. When CXCL10 levels were analyzed in the serum, significantly increased concentrations were found at 14 dpi, coinciding with initial parasite neuroinvasion. This response appeared to be delayed in the CSF, and augmented CXCL10 levels were detected in the CSF at 21 dpi complementing the ongoing neuroinflammatory reaction. Furthermore the analysis of sleep/wake changes showed an initial fragmentation of vigilance states, especially during the period of wakefulness predominance, at the time of parasite neuroinvasion, when SOREM episodes, whose onset preceded this event, showed a significant increase (Fig 6). The present data on the timing of parasite penetration into the brain parenchyma is in agreement with our previous findings [7, 21], and the observation of parasites crossing blood vessels to enter the neuropil further supports parasite traversal of the BBB as main pathogenetic event of this brain infection [7, 21].

The increase of *Ifn-γ* gene expression in the brain, here found between 14 and 21 dpi, is supported by data in a more chronic rat model of *T*. *b*. *brucei* infection with a course of two months, in which *Ifn-γ* mRNA levels showed a small increase between 22 and 35 dpi in the choroid plexus and peaked throughout the brain at 43 dpi [35]. Though of pathogenetic relevance, IFN-*γ* was not identified as an effective marker when its level was measured in the CSF of *T*. *b*. *gambiense* HAT patients [15].

The IFN-dependent chemokines CXCL9, CXCL10 and CXCL11 bind to a common receptor, CXCR3, which is expressed by activated T-cells, and the role of CXCL10 is especially critical for the recruitment of activated T-cells to the central nervous system in a variety of diseases [36]. In the brain, both neurons and glia can express *Cxcl10* [36] and astrocytes contribute to the production of CXCL10 in African trypanosomiasis [16]. The present data on increases of *Cxcl10* gene expression in the brain as well as of the level of this chemokine in the CSF during *T*. *b*. encephalitis in rats are in agreement with those previously obtained in a murine model, in which differential gene expression was identified using microarray analysis. In this study, increased *Cxcl9* expression was detected from 6 to 28 dpi, and *Cxcl11* transcript levels could not be examined since the mouse strain used in this model is a natural null mutant for this chemokine [16]. In the present rat model, the modest increase of *Cxcl9* and *Cxcl11* mRNAs coinciding with the time of initial parasite neuroinvasion could point to these chemokines as candidate CSF biomarkers of the onset of the encephalitic stage. A limitation of the present study is that the level of these chemokines in the CSF during the infection could not be measured due to the restricted sample volume available and thus remains to be assessed in future studies.

The changes in the concentration of CXCL10 in the serum and CSF here identified could reflect the systemic and brain progression of *T*. *b*. infection, respectively. During systemic inflammation CXCL10 is produced by monocytes and endothelial cells in a variety of pathologies, which include autoimmune diseases [37], inflammatory bowel diseases [38], chronic liver diseases and especially hepatitis C [39, 40]. Therefore CXCL10 levels in the serum may not provide specific information with regard to disease progression in HAT, although the present study suggests that increases of circulating CXCL10 levels in HAT patients should be carefully considered. Concerning the CSF, increases of CXCL10 have been demonstrated in other CNS infections, such as encephalitis caused by viruses and cerebral malaria [36, 41, 42]. The combination of neurological signs characteristic of HAT with high CXCL10 level in the CSF could therefore be of particular diagnostic relevance for the evaluation of the severity of this disease, especially *T*. *b*. *gambiense* HAT. Even in *T*. *b*. *rhodesiense* HAT, in which no direct link was found between neurological signs and neuroinflammatory responses [43], CXCL10 level was found to increase in the CSF in late stage disease [44], and CXCL10 has been utilized in combinatorial panels with potential efficacy for *T*. *b*. *rhodesiense* HAT staging [19].

Two aspects of vigilance states, sleep structure and sleep/wake cycle, were here examined. Concerning the first, the present findings support previous data indicating that the onset of SOREM episodes can precede parasite neuroinvasion [21]. Concerning the changes in the sleep/wake cycle, an increase in the number of state transitions indicative of sleep/wake fragmentation, associated with a decrease of the mean duration of W episodes, was found at the time of parasite neuroinvasion during the phase of wake predominance. This was accompanied by an initial phase inversion, with intrusion of sleep into wakefulness. The findings indicate that a disruption of the sleep/wake pattern is likely associated with the initial encephalitic stage, as supported by previous experimental studies [24–26], here extended to include an assessment of parasites in the brain parenchyma, and parallel gene expression and CXCL10 analyses.

Sleep/wake changes documented in African trypanosomiasis are different from those of occurring during systemic infections, which are part of the so-called “sickness response” and are characterized by SWS increase [45], and from those occurring during other cerebral infections [46]. Concerning pathogenetic mechanisms, cytokine effects on the master circadian pacemaker, the hypothalamic suprachiasmatic nucleus, could account in African trypanosomiasis for disturbances of the sleep/wake cycle [7], as well as for phase advance of the synchronizer hormone melatonin, whose rhythm of secretion is, however, maintained in HAT patients [47] and the rat model of the infection [48]. Factors secreted by African trypanosomes, such as prostaglandin D2, could affect vigilance states [8]. However, prostaglandin D2 is a potent endogenous sleep-promoting substance [49], whose effect cannot, therefore, account for the distinct changes of the sleep/wake cycle here observed.

Of note, the early meningoencephalitic stage examined in this study corresponds to a temporal window especially critical for therapeutic decisions. In *T*. *b*. *brucei*-infected mice, treatment with the early stage drug Suramin was found to exert a curative effect in the initial phase of parasite neuroinvasion, but was no longer effective at 21 dpi [50]. An objective evaluation of sleep/wake changes could, therefore, be of special interest for translational implications when the disease could still be susceptible to treatment with trypanocidal drugs which poorly cross the BBB. Sleep/wake fragmentation can be objectively documented in humans by the non-invasive and user-friendly procedure of actimetric recordings [51, 52], as verified also in *T*. *b*. *gambiense* HAT patients [53].

In conclusion, the present findings indicate that the onset of sleep/wake fragmentation during 24 h, and in particular episodes of somnolence during the day, together with an increase of CXCL10 levels in the serum and followed by an increase of CXCL10 level in the CSF, could provide a sensitive biomarker for initial African trypanosome neuroinvasion. Therefore, sleep/wake changes together with CXCL10 level in the CSF, by itself or in a combinatorial approach with other neuroinflammatory markers, could be highly relevant for a diagnosis of the early encephalitic stage of *T*. *b*. infection.

## Supporting information

S1 Table Rats were infected with *T*. *b*. *brucei* and killed at 0, 6, 14 and 21 days post-infection (dpi). RT-PCR reaction products for each sample from discrete time points and gene of interest were electrophoresed through a single 2% agarose gel and the corresponding band intensities quantified using an Uvitec gel documentation system. The resulting densitometry data was then normalised against *Cyclophilin* expression. The figures in the body of the table demonstrate the comparisons, in terms of statistical significance, between the time points shown in the row and column headings for each gene of interest. The *p-*values and 95% confidence intervals are based on ANOVA followed by Tukey’s *post-hoc* analysis. The mean band intensity ± the standard error and the number of rats per group are also shown.
(DOCX)
