# Supplementary material for: Expression of interferon-inducible chemokines and sleep/wake changes during early encephalitis in experimental African trypanosomiasis
Source: PLoS Negl Trop Dis. 2017 Aug 18;11(8):e0005854. doi: 10.1371/journal.pntd.0005854 (PMC5576758; doi:10.1371/journal.pntd.0005854)
Supplement: S1 Table — RT-PCR reaction products for each sample from discrete time points and gene of interest were electrophoresed through a single 2% agarose gel and the corresponding band intensities quantified using an Uvitec gel documentation system. The resulting densitometry data was then normalised against Cyclophilin expression. The figures in the body of the table demonstrate the comparisons, in terms of statistical significance, between the time points shown in the row and column headings for each gene of interest. The p-values and 95% confidence intervals are based on ANOVA followed by Tukey’s post-hoc analysis. The mean band intensity ± the standard error and the number of rats per group are also shown. (DOCX) [file pntd.0005854.s001.docx]

| ***Cxcl*9** | 0 dpi | 6 dpi | 14 dpi | 21 dpi |
| --- | --- | --- | --- | --- |
| 6 dpi | *p*=0.594  (-0.140, 0.328) |  |  |  |
| 14 dpi | *p*=0.001  (0.224, 0.691) | *p*=0.005  (0.130, 0.597) |  |  |
| 21 dpi | *p*<0.001  (1.336, 1.803) | *p*<0.001  (1.242, 1.709) | *p*<0.001  (0.878, 1.346) |  |
| Mean + SE  Number of rats | 0.054 + 0.024  3 | 0.148 + 0.064  3 | 0.511 + 0.058  3 | 1.623 + 0.051  3 |

| ***Cxcl*10** | 0 dpi | 6 dpi | 14 dpi | 21 dpi |
| --- | --- | --- | --- | --- |
| 6 dpi | *p*=0.993  (-0.545, 0.461) |  |  |  |
| 14 dpi | *p*=0.981  (-0.444, 0.562) | *p*=0.914  (-0.402, 0.604) |  |  |
| 21 dpi | *p*<0.001  (1.148, 2.154) | *p*<0.001  (1.191, 2.197) | *p*<0.001  (1.089, 2.095) |  |
| Mean + SE  Number of rats | 0.259 + 0.036  3 | 0.217 + 0.029  3 | 0.318 + 0.74  3 | 1.911 + 0.204  3 |

| ***Cxcl*11** | 0 dpi | 6 dpi | 14 dpi | 21 dpi |
| --- | --- | --- | --- | --- |
| 6 dpi | *p*=0.178  (-0.149, 0.907) |  |  |  |
| 14 dpi | *p*=0.004  (0.334, 1.390) | *p*=0.073  (-0.045, 1.011) |  |  |
| 21 dpi | *p*<0.001  (1.961, 3.017) | *p*<0.001  (1.582, 2.638) | *p*<0.001  (1.099, 2.155) |  |
| Mean + SE  Number of rats | 0.182 + 0.026  3 | 0.561 + 0.082  3 | 1.044 + 0.109  3 | 2.671 + 0.187  3 |

| ***Ifn*γ** | 0 dpi | 6 dpi | 14 dpi | 21 dpi |
| --- | --- | --- | --- | --- |
| 6 dpi | *p*=0.439  (-0.265, 0.781) |  |  |  |
| 14 dpi | *p*=0.051  (-0.002, 1.044) | *p*=0.426  (-0.260, 0.786) |  |  |
| 21 dpi | *p*<0.001  (1.497, 2.543) | *p*<0.001  (1.239, 2.285) | *p*<0.001  (0.977, 2.023) |  |
| Mean + SE  Number of rats | 0.244 + 0.052  3 | 0.502 + 0.075  3 | 0.765 + 0.108  3 | 2.264 + 0.182  3 |

**Supplementary Table 1.** Rats were infected with *T. b. brucei* and killed at 0, 6, 14 and 21 days post-infection (dpi). RT-PCR reaction products for each sample from discrete time points and gene of interest were electrophoresed through a single 2% agarose gel and the corresponding band intensities quantified using an Uvitec gel documentation system. The resulting densitometry data was then normalised against *Cyclophilin* expression. The figures in the body of the table demonstrate the comparisons, in terms of statistical significance, between the time points shown in the row and column headings for each gene of interest. The *p-*values and 95% confidence intervals are based on ANOVA followed by Tukey’s *post-hoc* analysis. The mean band intensity + the standard error and the number of rats per group are also shown.
